# Propofol Augments Paclitaxel-Induced Cervical Cancer Cell Ferroptosis *In Vitro*

**DOI:** 10.3389/fphar.2022.816432

**Published:** 2022-04-20

**Authors:** Meng-Yun Zhao, Pan Liu, Chen Sun, Li-Jian Pei, Yu-Guang Huang

**Affiliations:** ^1^ Department of Anesthesiology, Peking Union Medical College Hospital, Chinese Academy of Medical Sciences, Beijing, China; ^2^ Joint Laboratory of Anesthesia and Pain, Peking Union Medical College, Beijing, China; ^3^ Department of Human Anatomy, Histology and Embryology, Institute of Basic Medical Sciences, Chinese Academy of Medical Sciences, School of Basic Medicine, Peking Union Medical College, Beijing, China; ^4^ Department of Hematology, Zhongnan Hospital of Wuhan University, Wuhan, China; ^5^ Outcomes Research Consortium, Cleveland, OH, United States

**Keywords:** propofol, paclitaxel, cervical cancer, ferroptosis, apoptosis

## Abstract

**Introduction:** Cervical cancer is common in women. The present standardized therapies including surgery, chemotherapy, and radiotherapy are still not enough for treatment. Propofol is the most commonly used intravenous anesthetic agent for induction and maintenance of anesthesia and has been shown to exert anti-malignancy effects on cancer cells, inducing oxidative stress and apoptosis. However, the biological effects of propofol have not yet been systematically assessed. In this study, we examined the ferroptosis-related changes caused by propofol and the chemotherapeutic agent paclitaxel besides apoptosis *in vitro*.

**Methods:** Cervical cancer cell lines (C-33A and HeLa) were treated with propofol alone (1, 2, 5, 10, and 20 μg/ml) or in combination with paclitaxel (0.5, 1, and 5 μg/ml). The viability was assessed using cell counting kit-8 (CCK8), apoptosis was detected by flow cytometry, morphological changes of mitochondria were examined using transmission electron microscope (TEM), cellular reactive oxygen species (ROS), and intracellular ferrous ions were determined by fluorescence microscope or confocal microscopy. The expression and cellular localization of apoptosis and ferroptosis-related molecules were detected by Western blot and multiplex immunohistochemistry (mIHC), respectively. Calcusyn software was used to determine whether propofol has a synergistic effect with paclitaxel.

**Results:** Propofol and paclitaxel inhibited C-33A and HeLa cell viability. There were also synergistic effects when propofol and paclitaxel were used in combination at certain concentrations. In addition, propofol promoted paclitaxel-induced cervical cancer cell death via apoptosis. ROS level and Fe^2+^ concentrations were also influenced by different drug treatments. Furthermore, propofol, propofol injectable emulsion, and paclitaxel induced ferroptosis-related morphological changes of mitochondria in C-33A and HeLa cells. Ferroptosis-related signaling pathways including SLC7A11/GPX4, ubiquinol/CoQ10/FSP1, and YAP/ACSL4/TFRC were found to be changed under drug treatments.

**Conclusion:** Propofol showed synergistic anticancer effects with paclitaxel in cervical cancer cells. Propofol and paclitaxel may induce ferroptosis of cervical cancer cells besides apoptosis.

## Introduction

Cervical cancer is a kind of common cancer in women (Bray et al., 2018). Standardized therapies involve surgical resection for early-stage cervical cancer and systemic chemotherapy in combination with radiotherapy for progressive cervical cancer (Olusola et al., 2019). However, even after curative primary resection, metastasis and recurrence are reported (Freeman et al., 2016). For anesthesiologists, it is of great interest to improve anesthetic management to reduce the cancer burden. The ways in which anesthetic management is conducted and the type of anesthetic drug chosen during the few hours of the anesthetic management period may influence cancer recurrence months or years later (Sessler and Riedel, 2019). To explore the potential effects of anesthetic drugs on cervical cancer cells is crucial.

Propofol (2,6-diisopropylphenol) is the most frequently used intravenous sedative–hypnotic agent for both induction and maintenance of anesthesia. Animal experiments and *in vitro* cell experiments consistently show that propofol possesses the potential to inhibit the malignancy of primary cancer (Sessler and Riedel, 2019). Several literature discussing relationships among propofol, chemotherapeutic agents, and cancer cell *in vitro* have focused on cell apoptosis (Li et al., 2017; Li et al., 2021; Shen et al., 2021). Cell apoptosis caused by propofol and chemotherapeutic drugs has been widely reported. To the best of our knowledge, none of the previous studies have reported the effects of propofol on cancer cell ferroptosis.

Ferroptosis is a unique form of regulated cell death (RCD), distinguishing itself from apoptosis, autophagy, and necrosis by characteristic iron-dependent accumulation of lipid hydroperoxides to lethal levels. Glutathione peroxidase 4 (GPX4) is one of the central regulating enzymes that protects cells from ferroptosis by neutralizing lipid peroxides, which are the byproducts of cellular metabolism (Stockwell et al., 2017; Wu et al., 2019). Ferroptosis can be initiated through two major pathways: the extrinsic pathway by inhibition of cell membrane transporters such as the cystine/glutamate transporter and the intrinsic pathway by blocking of intracellular antioxidant enzymes such as GPX4 (Chen et al., 2021). SLC7A11 is the cystine/glutamate transporter which acts as a suppressor for ferroptosis (Koppula et al., 2021). FSP1/ubiquinone (Coenzyme Q10, or CoQ10) is a pathway against ferroptosis independent of GPX4. FSP1 functions as a suppressor of ferroptosis through CoQ10, and CoQ10 is an antioxidant that protects cells from lipid peroxidation. Ubiquinol, the reduced form of CoQ10, traps lipid peroxyl radicals that mediate lipid peroxidation (Doll et al., 2019; Tsui et al., 2019). Furthermore, the intracellular YAP/ACSL4/TFRC signaling also regulates the activities of ferroptosis (Wu et al., 2019).

Our study investigated whether propofol or PIE in combination with or without paclitaxel at clinical concentrations exerts effects on apoptosis and ferroptosis of C-33A and HeLa cells and also how different drug treatment regimens regulate pathways involved in ferroptosis including SLC7A11/GPX4, ubiquinol/CoQ10/FSP1, and YAP/ACSL4/TFRC. The apoptosis of cancer cells caused by anesthetic drugs and chemotherapeutic drugs has been widely reported. Partial common characteristics can be found in both apoptosis and ferroptosis, such as ROS accumulation (Su et al., 2019), but whether these drugs could cause ferroptosis in a certain portion of cancer cells besides apoptosis is still elusive, so we examined the ferroptosis-related features and found that drug treatment could cause ferroptosis-related features and treatment of anesthetic drugs in combination with chemotherapeutic drug-enhanced ferroptosis-related features of cervical cancer cells.

## Materials and Methods

### Cell Culture

Human cervical cancer cell line C-33A and HeLa were obtained from the Chinese National Infrastructure of Cell Line Resource (Beijing, China). C-33A and HeLa cells were cultured with Minimum Essential Medium (MEM) (11095080, Gibco, Grand Island, NY, United States) containing 1% nonessential amino acids (NEAA) (11140050, Gibco, Grand Island, NY, United States), and Roswell Park Memorial Institute (RPMI) 1640 medium (61870036, Gibco, Grand Island, NY, United States), respectively, supplemented with 10% fetal bovine serum (FBS) (AQmv09900, Analysis Quiz, Uruguay, South America) and 1% penicillin–streptomycin (15140122, Gibco, ThermoFisher Scientific) in an incubator with 5% CO_2_ at 37°C.

### Reagents

Propofol was purchased from Sigma-Aldrich (1572503, St. Louis, MO, United States). Propofol injectable emulsion (PIE) was obtained from AstraZeneca Co., United Kingdom. Paclitaxel was from Apexbio (A4393, Houston, TX, United States). All the reagents were dissolved according to the manufacturer’s instructions. Stock solutions of propofol and paclitaxel were stored in −20°C, while PIE was stored in 4°C.

### Cell Viability Assay

Before cell viability detection, propofol and PIE were administered to cells at the concentrations of 1, 2, 5, 10, and 20 μg/ml for 24 h. Paclitaxel was given at the concentrations of 0.5, 1, and 5 μg/ml for 24 h. The viability of C-33A and HeLa cells were detected by Cell Counting Kit-8 (CCK-8, Dojindo Laboratories, Japan). Briefly, the cells were seeded into 96-well plates (Costar, Corning, NY, United States) at 5 × 10^3^ cells per well. The cells were cultured overnight and then replenished with fresh medium containing drugs at indicated concentrations for 24 h. Before detection, the plates were replenished with fresh medium containing 10 µl of CCK-8 for each well and incubated for 2 h. The optical density (OD) was measured at 450 nm on an Epoch Microplate Reader (BioTek, Winooski, VT, United States). The viability of cells was calculated as cell viability (%) = (OD of treatment − OD of blank control)/(OD of control − OD of blank control) × 100%.

### Analysis of Cytotoxic Synergy

The combination index (CI) values were calculated using Calcusyn software to determine whether propofol or PIE has a synergistic effect with paclitaxel. The CI values were calculated as the following equation: CI = (D)1/(Dx)1 + (D)2/(Dx)2 + (D)1(D)2/(Dx)1(Dx)2, where (Dx)1 or (Dx)2 indicates the dosage for x% inhibition by drug 1 or drug 2 alone, (D)1 or (D)2 indicates the dosage in combination that inhibits cell growth by x%. A CI value of 1 suggests additive effects of the two drugs, while a CI value greater than 1 suggests antagonism effects, and a CI value less than 1 indicates synergism effects.

### Flow Cytometry

To detect the apoptosis of C-33A and HeLa cells, flow cytometry experiments were conducted using apoptosis detection kit (Dojindo Laboratories, Japan). After treatment with drugs for 24 h, C-33A and HeLa cells were digested with 0.05% trypsin (Gibco, ThermoFisher Scientific), and then collected and washed with ice cold PBS two times; 1× Annexin V Binding Solution was added to make a cell suspension with a concentration of 1 × 10^6^ cells/ml. The cells were then stained with Annexin V, fluorescein isothiocyanate (FITC), and propidium iodide (PI) for 15 min at room temperature (RT) before 400 µl 1× Annexin V Binding Solution was added. The cells were then loaded to a flow cytometer (Accuri C6 Plus, BD BioSciences, United States) within 1 h. Results were analyzed using BD Accuri C6 Plus software.

### Transmission Electron Microscope (TEM)

TEM was used to examine the mitochondrial morphological changes of C-33A and HeLa cells after drug treatment at indicated concentrations. C-33A and HeLa cells were trypsinized, harvested, and fixed in 2.5% glutaraldehyde (EM Grade) at 4°C overnight. After fixation in 1% osmium acid, cell samples were subsequently dehydrated, and placed in embedding molds in a standard fashion. Appropriate areas were selected and ultrathin sections of 0.08 µm were stained with lead citrate and uranyl acetate. Those sections were then examined using a transmission electron microscope (JEM-1400Plus, JEOL, Ltd., Tokyo, Japan).

### Cellular ROS Assay

C-33A and HeLa cells were treated with indicated concentrations. Cells were then washed two times with 1× buffer and stained by diluted DCFDA Solution (Abcam Plc., Cambridge, United Kingdom) for 45 min in an incubator. Then, the cells were washed two times with 1× buffer. The cells were observed using a fluorescent microscopy under low light conditions. Cellular ROS proportion were analyzed with Image J software and compared between the negative control (NC) and drug treated groups.

### FerroOrange Iron Assay

C-33A and HeLa cells were seeded into 8-well plates and incubated overnight. The cells were then replenished with fresh medium containing drugs at indicated concentrations and incubated for 24 h. Then, the cells were washed with serum-free medium 3 times; 1 μmol/l of FerroOrange working solution (Dojindo Laboratories, Japan) was added into each well and incubated for 30 min. The cells were imaged with a laser scanning confocal microscope (UltraVIEW VOX, PerkinElmer, Inc., Waltham, MA, United States). Relative mean fluorescence intensity (MFI) of ferrous ions was calculated using ImageJ software.

### Western Blot

C-33A and HeLa cells were treated with drugs at indicated concentrations for 24 h, followed by lysis in RIPA buffer containing protease inhibitor. Protein concentrations were determined using bicinchoninic acid assay system (Beyotime, Shanghai, China). Protein samples were added 20 µg per lane and separated by SDS-PAGE gel and electrophoretically transferred to polyvinylidene fluoride (PVDF) membrane. The membrane was then blocked with 5% skimmed milk for 2 h at RT. Primary antibodies including anti-Bcl-2 (Abcam, ab182858), anti-caspase-3 (Abcam, ab32351), anti-SLC7A11 (Abcam, ab175186), anti-GPX4 (Abcam, ab125066), anti-ubiquinol-cytochrome C reductase core (Abcam, ab110252), anti-CoQ10A (Proteintech, 17812-1-AP), anti-FSP1 (Proteintech, 20886-1-AP), anti-YAP (CST, 14074S), anti-ACSL4 (Abcam, ab155282), and anti-TFRC (Abcam, ab84036) antibodies were diluted in primary antibody dilution buffer (NCM, WB100D) and incubated with PVDF membrane at 4°C overnight. Next, the membrane was incubated with goat anti-mouse/anti-rabbit secondary antibodies (Proteintech, SA00001-1, SA00001-2) at RT for 2 h, followed by detection via an enhanced chemiluminescence detection kit (New Cell and Molecular, P10100). β-Actin (Proteintech, 66009-1-Ig) was used as the internal control. Images were captured via a chemiluminescence imaging system (Tanon 5800, Shanghai, China).

### Multiplex Immunohistochemistry (mIHC)

Multiplex immunohistochemistry modified for adherent C-33A and HeLa cells was performed to identify the protein expression and localization using the PANO Multiplex IHC Kit (0001100100, Panovue, Beijing, China).

### Statistical Analysis

Statistical analysis was conducted by performing one-way ANOVA followed with Tukey’s post hoc analysis using GraphPad Prism 8.1.1 software to compare the differences between two groups, and data were presented as mean ± standard deviation. A *p* value of <0.05 was considered to be statistically significant.

## Results

### Propofol/PIE Inhibits Cell Viability and Enhances Sensitivity of Paclitaxel for Cervical Cancer Cell *In Vitro*

**FIGURE 1:**
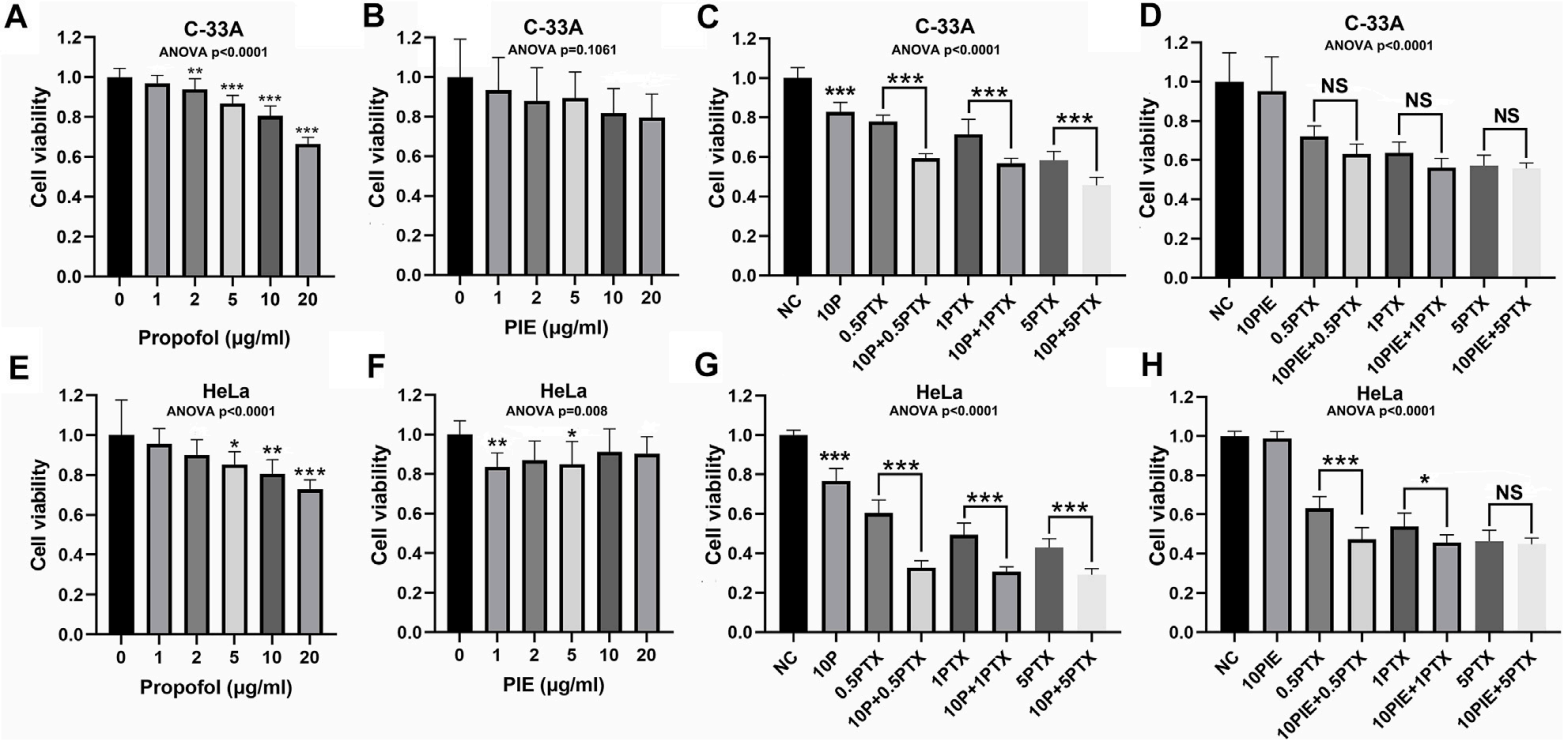
Effects of propofol or PIE with or without paclitaxel on cell viability of C-33A and HeLa cells. **(A,B)** Effects of propofol/PIE on cell viability of C-33A cells. **(C,D)** Combination effects of propofol/PIE with paclitaxel on cell viability of C-33A cells. **(E,F)** Effects of propofol/PIE on cell viability of HeLa cells. **(G,H)** Combination effects of propofol/PIE with paclitaxel on cell viability of HeLa cells. NS, no significant difference. *p < 0.05, **p < 0.01, ***p < 0.001. 10P: 10 μg/ml propofol; 0.5PTX: 0.5 μg/ml paclitaxel; 10P+0.5PTX: 10 μg/ml propofol plus 0.5 μg/ml paclitaxel; 1PTX: 1 μg/ml paclitaxel; 10P+1PTX: 10 μg/ml propofol plus 1 μg/ml paclitaxel; 5PTX: 5 μg/ml paclitaxel; 10P+5PTX: 10 μg/ml propofol plus 5 μg/ml paclitaxel; 10PIE: 10 μg/ml PIE; 10PIE+0.5PTX: 10 μg/ml PIE plus 0.5 μg/ml paclitaxel; 10PIE+1PTX: 10 μg/ml PIE plus 1 μg/ml paclitaxel; 10PIE+5PTX: 10 μg/ml PIE plus 5 μg/ml paclitaxel.

Cervical cancer cell lines C-33A and HeLa cells were treated with 1–20 μg/ml propofol or PIE, 0.5–5 μg/ml paclitaxel, or 10 μg/ml propofol or PIE combined with 0.5–5 μg/ml paclitaxel for 24 h. In C-33A cells, exposure to 2, 5, 10, or 20 μg/ml propofol for 24 h resulted in significant decrease of cell viability compared with NC (*p* < 0.01), however, PIE did not significantly reduce C-33A cell viability compared to NC (Figures 1A,B). In combination treatment groups of C-33A cells, exposure to 10 μg/ml propofol combined with paclitaxel for 24 h resulted in significant decrease of cell viability compared with treatment of paclitaxel alone (*p* < 0.001); however, 10 μg/ml PIE combined with paclitaxel did not significantly inhibit C-33A cell viability compared to paclitaxel treatment alone (Figures 1C,D). Propofol at 10 μg/ml significantly reduced the C-33A cell viability by 19.44% (*p* < 0.001), while 10 μg/ml of PIE did not significantly decrease the C-33A cell viability. Propofol at 10 μg/ml, when added to 5 μg/ml of paclitaxel, produced a further significant reduction in the C-33A cell viability of 12.79% (*p* < 0.001), whereas PIE at 10 μg/ml added to a similar concentration of paclitaxel had no significant effect. The CI values of the co-administration of propofol with paclitaxel in C-33A cells were 0.460, 0.484, and 0.465 corresponding to 10P+0.5PTX, 10P+1PTX, and 10P+5PTX groups. The CI values were 0.235, 0.197, and 0.803 when PIE and paclitaxel were co-administered in C-33A cells corresponding to the groups of 10PIE+0.5PTX, 10PIE+1PTX, and 10PIE+5PTX, respectively. Thus, both propofol and PIE in combination of paclitaxel can exert cytotoxic synergistic effects to inhibit C-33A cell viability.

In HeLa cells, exposure to 5, 10, or 20 μg/ml propofol, or 1 or 5 μg/ml PIE for 24 h significantly reduced cell viability compared with NC (*p* < 0.05, Figures 1E,F). For combination treatment, 10 μg/ml propofol or PIE combined with 0.5 or 1 μg/ml paclitaxel for 24 h significantly decreased HeLa cell viability compared with the same concentration of paclitaxel alone (*p* < 0.05, Figures 1G,H). Propofol at 10 μg/ml significantly inhibited HeLa cell viability by 19.40% (*p* < 0.01), while 10 μg/ml of PIE did not significantly decrease HeLa cell viability. Propofol at 10 μg/ml, when added to 5 μg/ml of paclitaxel, produced a further significant reduction in the HeLa cell viability of 13.66% (*p* < 0.001), whereas PIE at 10 μg/ml added to a similar concentration of paclitaxel showed no significant effect. In HeLa cells, when propofol and paclitaxel were administered to the groups of 10P+0.5PTX, 10P+1PTX, and 10P+5PTX; the CI values were 0.065, 0.065, and 0.168, respectively. When PIE and paclitaxel were co-administered in HeLa cells to the groups of 10PIE+0.5PTX, 10PIE+1PTX, and 10PIE+5PTX; the CI values were all greater than 1, which means antagonistic effects of PIE with paclitaxel. Thus, for HeLa cells, propofol but not PIE has a synergistic effect on the inhibition of cell viability when combined with paclitaxel.

As above, our data suggest that propofol is a potential adjuvant to augment the inhibitory effects of paclitaxel on viability of cervical cancer cells. However, PIE showed different properties compared with propofol.

### Propofol/PIE Induces Apoptosis and Enhances Sensitivity of Paclitaxel-Induced Apoptosis for Cervical Cancer Cell *In Vitro*

**FIGURE 2:**
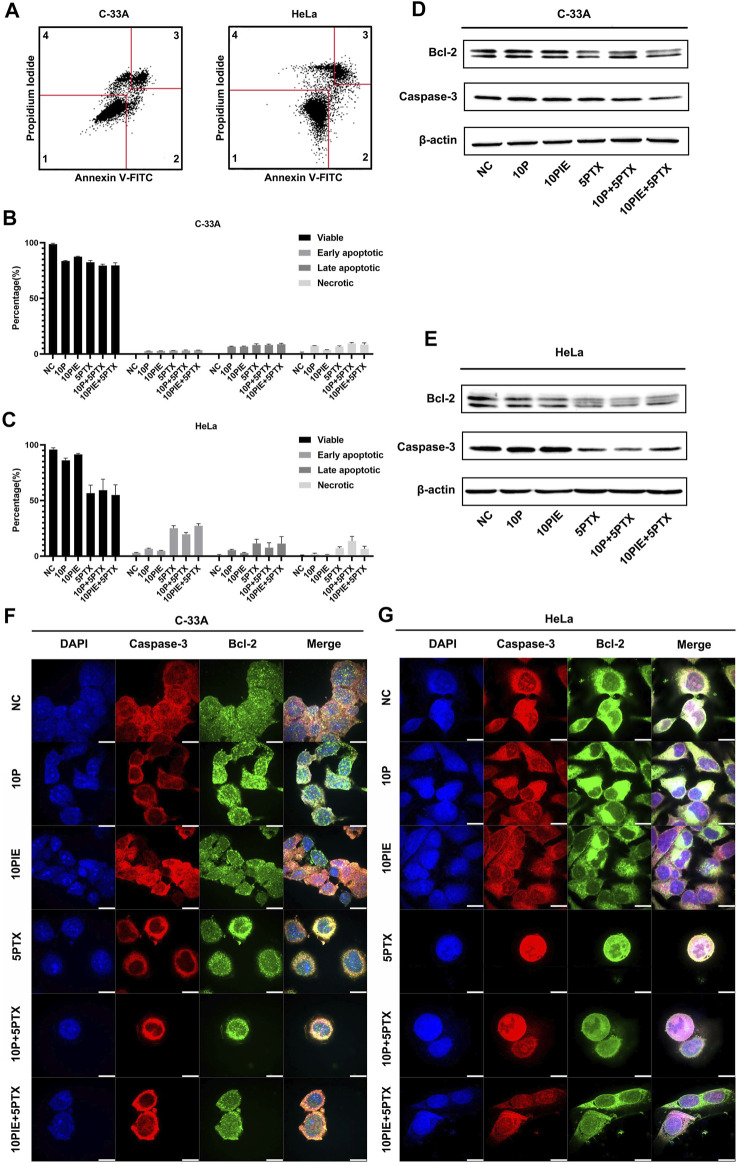
Effects of propofol or PIE with or without paclitaxel on cervical cancer cell apoptosis. **(A)** Gating strategy for cell apoptosis assessment. Representative plots were obtained from 5 μg/ml paclitaxel-treated C-33A cells and 5 μg/ml paclitaxel-treated HeLa cells. **(B,C)** Percentage of viable, early apoptotic, late apoptotic, and necrotic C-33A and HeLa cells treated with propofol/PIE with or without paclitaxel. **(D,E)** The expression level of Bcl-2 and caspase-3 in C-33A and HeLa cells under treatment of different drug combinations. β-Actin was used as the internal reference. **(F,G)** The expression and subcellular localization of Bcl-2 and caspase-3 in C-33A and HeLa cells after treatment with different drug combinations were indicated by mIHC. DAPI was used to stain the nuclei of the cells. All the bars indicated 10 µm.

To assess whether the effects of propofol or PIE on the viability of cervical cancer cells were correlated with cell apoptosis, we conducted flow cytometry experiment for apoptosis analysis (Figures 2A–C). Western blot analysis and mIHC of adherent C-33A and HeLa cells were also performed to identify the potential molecular mechanisms. We found that the drug treatments caused apoptosis of cervical cancer cells, which could explain the inhibition of cell viability caused by drug treatments from a certain perspective.

In C-33A cells, the percentage of early apoptotic cells significantly increased after single drug treatment with 10 μg/ml propofol [(2.60 ± 0.10%) vs. (0.50 ± 0.20%), *p* < 0.001] or PIE [(2.67 ± 0.15%) vs. (0.50 ± 0.20%), *p* < 0.001], or 5 μg/ml paclitaxel [(3.03 ± 0.06%) vs. (0.50 ± 0.20%), *p* < 0.001] compared to NC. Combined treatment of 10 μg/ml propofol or PIE with 5 μg/ml paclitaxel did not significantly increase early apoptotic proportion of C-33A cells compared with paclitaxel treatment alone [(3.13 ± 0.38%) vs. (3.03 ± 0.06%), *p* = 0.99; (3.37 ± 0.12%) vs. (3.03 ± 0.06%), *p* = 0.36; respectively]. As for late apoptosis in C-33A cells, 10 μg/ml propofol [(6.57 ± 0.35%) vs. (0.00 ± 0.00%), *p* < 0.001] or PIE [(6.50 ± 0.53%) vs. (0.00 ± 0.00%), *p* < 0.001], or 5 μg/ml paclitaxel [(8.10 ± 1.06%) vs. (0.00 ± 0.00%), *p* < 0.001] could significantly increase late apoptotic percentage compared to NC. However, 10 μg/ml propofol [(8.07 ± 0.70%) vs. (8.10 ± 1.06%), *p* > 0.99] or PIE [(8.67 ± 0.86%) vs. (8.10 ± 1.06%), *p* = 0.90] combined with 5 μg/ml paclitaxel did not significantly increase late apoptotic C-33A cell proportion compared with paclitaxel treatment alone. Notably, 10 μg/ml propofol [(7.23 ± 0.21%) vs. (0.77 ± 0.93%), *p* < 0.001], 10 μg/ml PIE [(3.50 ± 0.26%) vs. (0.77 ± 0.93%), *p* < 0.05], or 5 μg/ml paclitaxel [(6.57 ± 0.64%) vs. (0.77 ± 0.93%), *p* < 0.001] also significantly increased the necrotic percentage of C-33A cells compared to NC; 10 μg/ml propofol plus 5 μg/ml paclitaxel [(9.47 ± 0.70%) vs. (6.57 ± 0.64%), *p* < 0.05] significantly increased the necrotic proportion of C-33A cells compared to 5 μg/ml paclitaxel treatment, while 10 μg/ml PIE plus 5 μg/ml paclitaxel did not show this significant enhancement [(8.47 ± 1.48%) vs. (6.57 ± 0.64%), *p* = 0.12] (Figure 2B).

For HeLa cells, the proportion of early apoptotic cells significantly increased after single drug treatment with 5 μg/ml paclitaxel [(25.07 ± 2.29%) vs. (3.33 ± 1.46%), *p* < 0.001]. However, treatment of 10 μg/ml propofol [(6.63 ± 0.51%) vs. (3.33 ± 1.46%), *p* = 0.16], or 10 μg/ml PIE [(4.70 ± 0.46%) vs. (3.33 ± 1.46%), *p* = 0.88] did not significantly increase early apoptotic HeLa cell proportion. Combined treatment of 10 μg/ml propofol with 5 μg/ml paclitaxel significantly decreased early apoptotic HeLa cell percentage compared with paclitaxel treatment alone [(19.63 ± 1.57%) vs. (25.07 ± 2.29%), *p* < 0.01]; 10 μg/ml PIE plus 5 μg/ml paclitaxel did not significantly affect the early apoptotic percentage compared to paclitaxel alone [(27.23 ± 1.99%) vs. (25.07 ± 2.29%), *p* = 0.54]. Late apoptotic proportion significantly elevated after 5 μg/ml paclitaxel [(11.40 ± 3.72%) vs. (0.07 ± 0.06%), *p* < 0.05] treatment. However, treatment of 10 μg/ml propofol [(5.40 ± 0.66%) vs. (0.07 ± 0.06%), *p* = 0.46], or 10 μg/ml PIE [(2.77 ± 0.49%) vs. (0.07 ± 0.06%), *p* = 0.92] did not significantly increase late apoptotic HeLa cell percentage.10 μg/ml propofol [(7.63 ± 4.39%) vs. (11.40 ± 3.72%), *p* = 0.76] or PIE [(11.30 ± 6.20%) vs. (11.40 ± 3.72%), *p* > 0.99] plus 5 μg/ml paclitaxel did not show significant effect on the late apoptotic proportion of HeLa cells compared with paclitaxel alone. For cell necrosis, 10 μg/ml propofol [(1.83 ± 0.83%) vs. (0.83 ± 0.15%), *p* > 0.99], or 10 μg/ml PIE [(1.10 ± 0.44%) vs. (0.83 ± 0.15%), *p* > 0.99] did not significantly increase necrotic HeLa cell proportion; 10 μg/ml propofol [(13.57 ± 7.60%) vs. (7.00 ± 1.35%), *p* = 0.22] or 10 μg/ml PIE [(6.57 ± 2.34%) vs. (7.00 ± 1.35%), *p* > 0.99] plus 5 μg/ml paclitaxel did not significantly increase necrotic proportion compared to paclitaxel alone (Figure 2C).

In C-33A cells, the anti-apoptotic protein Bcl-2 decreased in the groups of 5PTX, 10P+5PTX, and 10PIE+5PTX. Full-length caspase-3 also decreased notably in the groups of 10P+5PTX, 10PIE+5PTX (Figure 2D). In HeLa cells, the anti-apoptotic protein Bcl-2 decreased after drug treatment compared with NC, and caspase-3 decreased in groups involving paclitaxel treatment (Figure 2E). The expression and localization of caspase-3 and Bcl-2 in C-33A and HeLa cells were indicated in mIHC pictures (Figures 2F,G). Caspase-3 was mainly expressed in cytoplasm as indicated. Bcl-2 has a wider distribution range as indicated in Figures 2F,G.

### Propofol/PIE and Paclitaxel Induces Ferroptosis-Related Morphological Changes in C-33A and HeLa Cells

**FIGURE 3:**
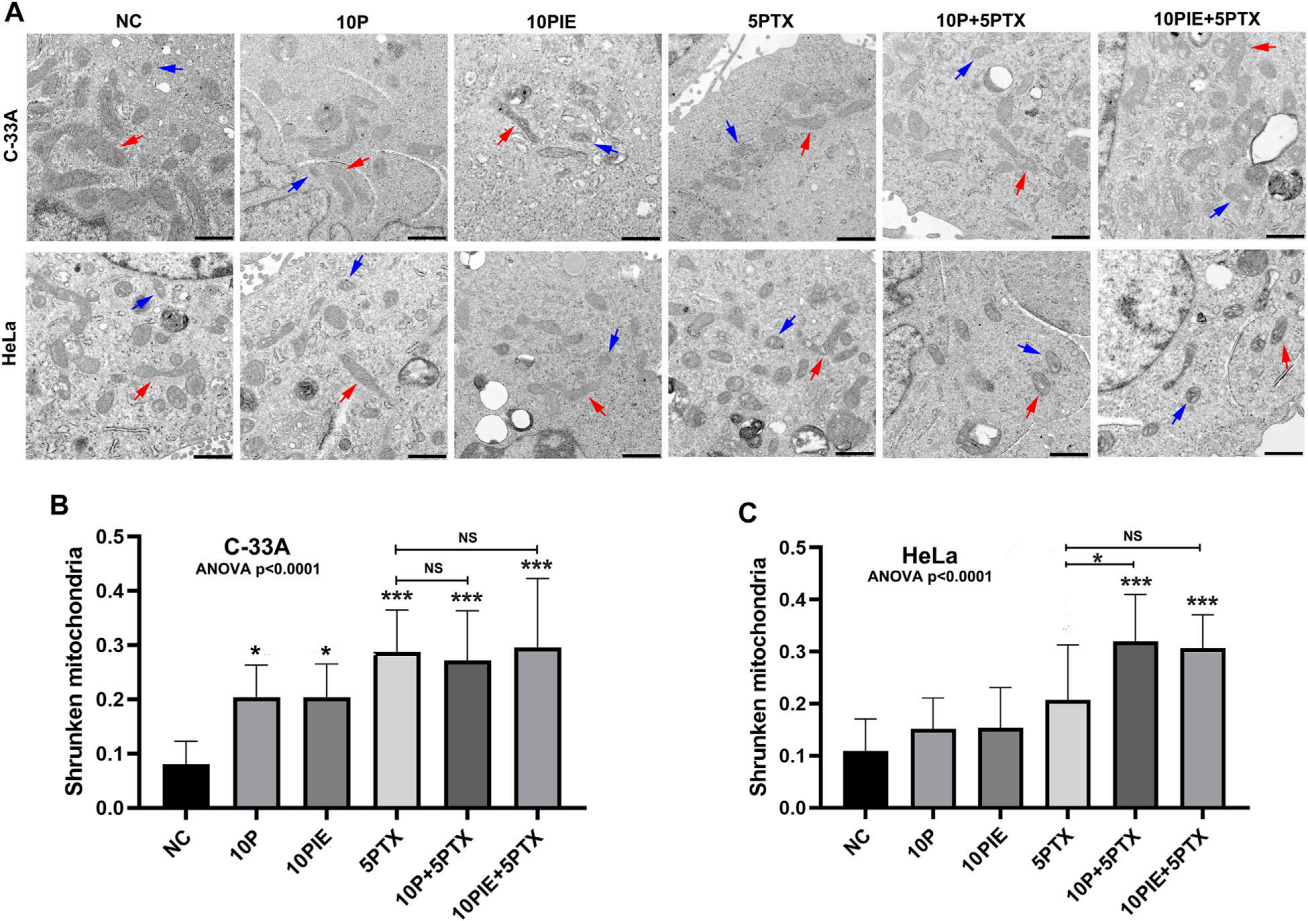
Propofol/PIE induced and enhanced paclitaxel-induced ferroptosis-related mitochondrial morphological changes of C-33A and HeLa cells. **(A)** TEM assay was conducted to determine mitochondrial morphological changes of ferroptosis in C-33A and HeLa cells. Red arrows: typical mitochondria morphology. Blue arrows: shrunken mitochondria morphology. All the bars indicated 1 µm. **(B,C)** Statistical analysis of shrunken mitochondria percentage in C-33A and HeLa cells under different drug treatments. NS, no significant difference. *p < 0.05, ***p < 0.001.

Through TEM examination, treatment of either 10 μg/ml propofol or PIE alone, or combined treatment of 10 μg/ml propofol/PIE with 5 μg/ml paclitaxel, increased the characteristic morphological changes of ferroptosis in C-33A and HeLa cells (Figure 3A). Shrunken mitochondria (smaller volume, thicker membrane, lesser mitochondrial cristae, etc.) was reported to be the morphological features of ferroptosis (Dixon et al., 2012), and we detected these features in this study to determine the effects of drugs on cervical cancer cells. The quantification of shrunken mitochondria was performed manually by counting shrunken and normal mitochondria in TEM images and calculating their proportion. Propofol or PIE at 10 μg/ml significantly increased shrunken mitochondria percentage in C-33A cells compared with NC [(0.20 ± 0.06) vs. (0.08 ± 0.04), *p* < 0.05; (0.20 ± 0.06) vs. (0.08 ± 0.04), *p* < 0.05; respectively; Figure 3B]; 5 μg/ml paclitaxel also significantly increased shrunken mitochondria percentage in C-33A cells compared to NC [(0.29 ± 0.08) vs. (0.08 ± 0.04), *p* < 0.001, Figure 3B]. In C-33A cells, 10 μg/ml propofol or PIE plus 5 μg/ml paclitaxel did not significantly increase ferroptosis-related morphological changes compared with the treatment of paclitaxel alone [(0.27 ± 0.09) vs. (0.29 ± 0.08), *p* > 0.99; (0.30 ± 0.13) vs. (0.29 ± 0.08), *p* > 0.99; respectively; Figure 3B]. In HeLa cells, neither 10 μg/ml propofol nor PIE caused any significant increase in the shrunken mitochondria percentage compared to NC [(0.15 ± 0.06) vs. (0.11 ± 0.06), *p* = 0.85; (0.15 ± 0.08) vs. (0.11 ± 0.06), *p* = 0.82; respectively; Figure 3C]; 5 μg/ml paclitaxel either did not significantly increase the percentage of shrunken mitochondria compared to NC [(0.21 ± 0.10) vs. (0.11 ± 0.06), *p* = 0.10, Figure 3C]. For combined treatment in HeLa cells, 10 μg/ml propofol plus 5 μg/ml paclitaxel significantly increased ferroptosis-related morphological changes of mitochondria compared with 5 μg/ml paclitaxel alone [(0.32 ± 0.09) vs. (0.21 ± 0.10), *p* < 0.05, Figure 3C], whereas 10 μg/ml PIE plus 5 μg/ml paclitaxel did not significantly increase shrunken mitochondria percentage compared to treatment of 5 μg/ml paclitaxel alone [(0.31 ± 0.06) vs. (0.21 ± 0.10), *p* = 0.09, Figure 3C].

These data demonstrate that propofol or PIE could induce ferroptosis-related morphological changes and increase paclitaxel-triggered intracellular ferroptosis-related morphological changes of cervical cancer cells *in vitro*.

### Propofol/PIE Plus Paclitaxel Combination Treatment Increases Cellular ROS Proportion of Cervical Cancer Cell

**FIGURE 4:**
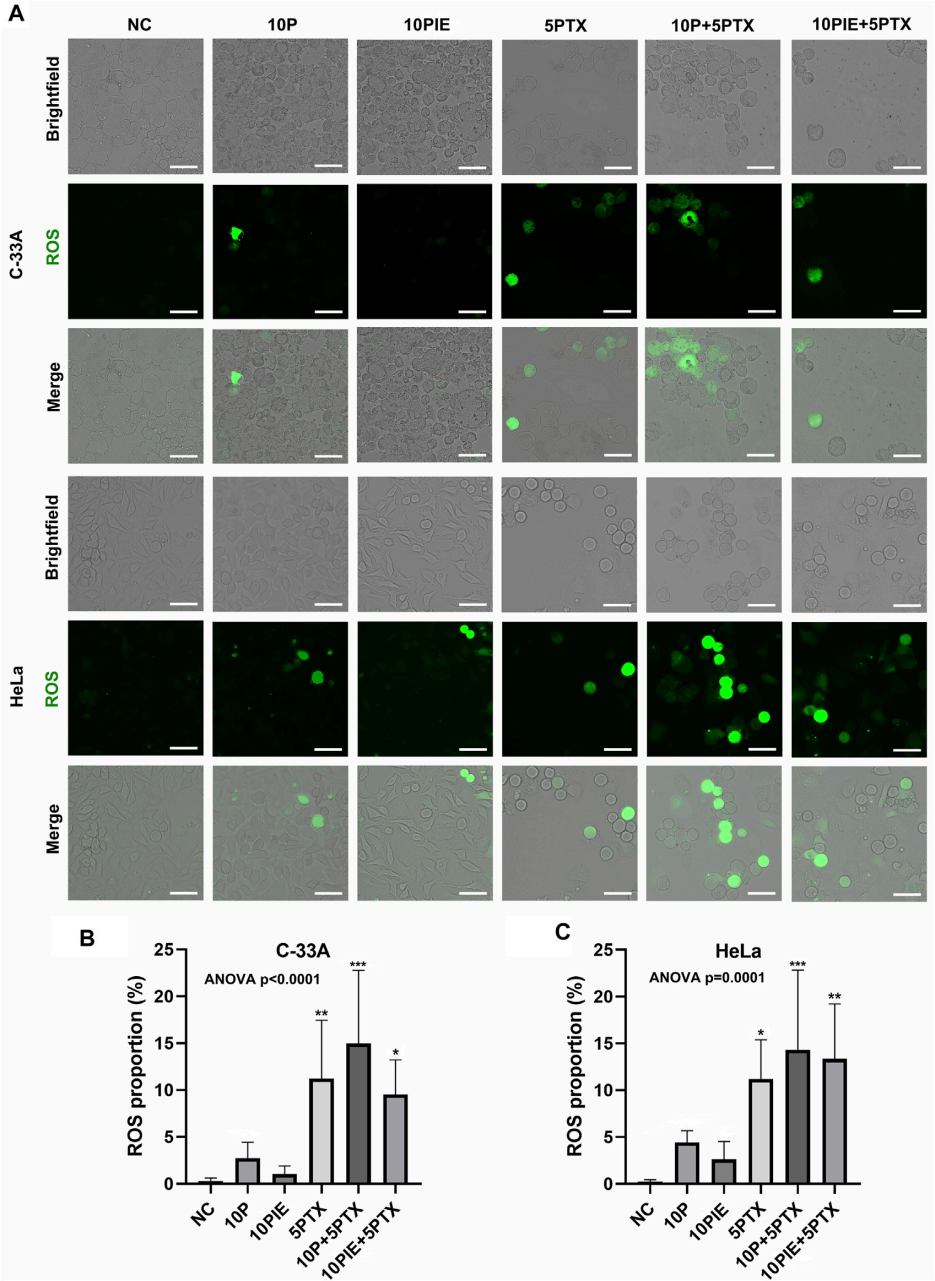
Propofol/PIE plus paclitaxel enhanced cellular ROS in C-33A and HeLa cells. **(A)** Intracellular ROS was detected via fluorescent microscopy and indicated as green spots. All the bars indicated 30 µm. **(B,C)** Statistical analysis of cellular ROS proportion in C-33A and HeLa cells after different combinations of drug treatment. *p < 0.05, **p < 0.01 and ***p < 0.001 represent statistical significance.

To identify the intracellular ROS level, fluorescent assay was adapted. As shown in Figure 4A, intracellular ROS-positive cells were increased after treatment of 10 μg/ml propofol, 10 μg/ml PIE, 5 μg/ml paclitaxel, 10 μg/ml propofol or PIE plus 5 μg/ml paclitaxel for 24 h.

In C-33A cells, the cellular ROS proportion (Figure 4B) was significantly increased after the combined treatment of 10 μg/ml propofol or PIE plus 5 μg/ml paclitaxel [(14.97 ± 7.80%) vs. (0.29 ± 0.32%), *p* < 0.001; (9.54 ± 3.69%) vs. (0.29 ± 0.32%), *p* < 0.05; respectively] compared to NC; 10 μg/ml propofol or PIE alone did not significantly enhance the cellular ROS proportion of C-33A cells [(2.75 ± 1.69%)vs. (0.29 ± 0.32%), *p* = 0.95; (1.04 ± 0.88%) vs. (0.29 ± 0.32%), *p* > 0.99; respectively] compared to NC. The combination drug treatment of 10 μg/ml propofol or PIE plus 5 μg/ml paclitaxel did not significantly enhance cellular ROS proportion in C-33A cells compared to 5 μg/ml paclitaxel [(14.97 ± 7.80%) vs. (11.22 ± 6.22%), *p* = 0.76; (9.54 ± 3.69%) vs. (11.22 ± 6.22%), *p* = 0.99; respectively].

For HeLa cells, the cellular ROS proportion (Figure 4C) significantly increased after the combined treatment of 10 μg/ml propofol or PIE plus 5 μg/ml paclitaxel [(14.31 ± 8.51%) vs. (0.25 ± 0.21%), *p* < 0.001; (13.35 ± 5.87%) vs. (0.25 ± 0.21%), *p* < 0.01; respectively] compared to NC. However, 10 μg/ml propofol [(4.43 ± 1.24%) vs. (0.25 ± 0.21%), *p* = 0.71], or 10 μg/ml PIE [(2.62 ± 1.91%) vs. (0.25 ± 0.21%), *p* = 0.96] did not significantly increase the cellular ROS proportion of HeLa cells compared to NC. Combined treatment of either 10 μg/ml propofol [(14.31 ± 8.51%) vs. (11.22 ± 4.18%), *p* = 0.89] or 10 μg/ml PIE [(13.35 ± 5.87%) vs. (11.22 ± 4.18%), *p* = 0.98] plus 5 μg/ml paclitaxel did not significantly increase the cellular ROS proportion compared to 5 μg/ml paclitaxel alone.

These findings indicate that drug treatment could enhance ROS accumulation in cervical cancer cells. Since ROS level was associated with both cell apoptosis and ferroptosis (Srinivas et al., 2019; Battaglia et al., 2020), we next examined the level of intracellular Fe^2+^ concentrations.

### PIE Increases Intracellular Fe^2+^ MFI in C-33A and HeLa Cells

**FIGURE 5:**
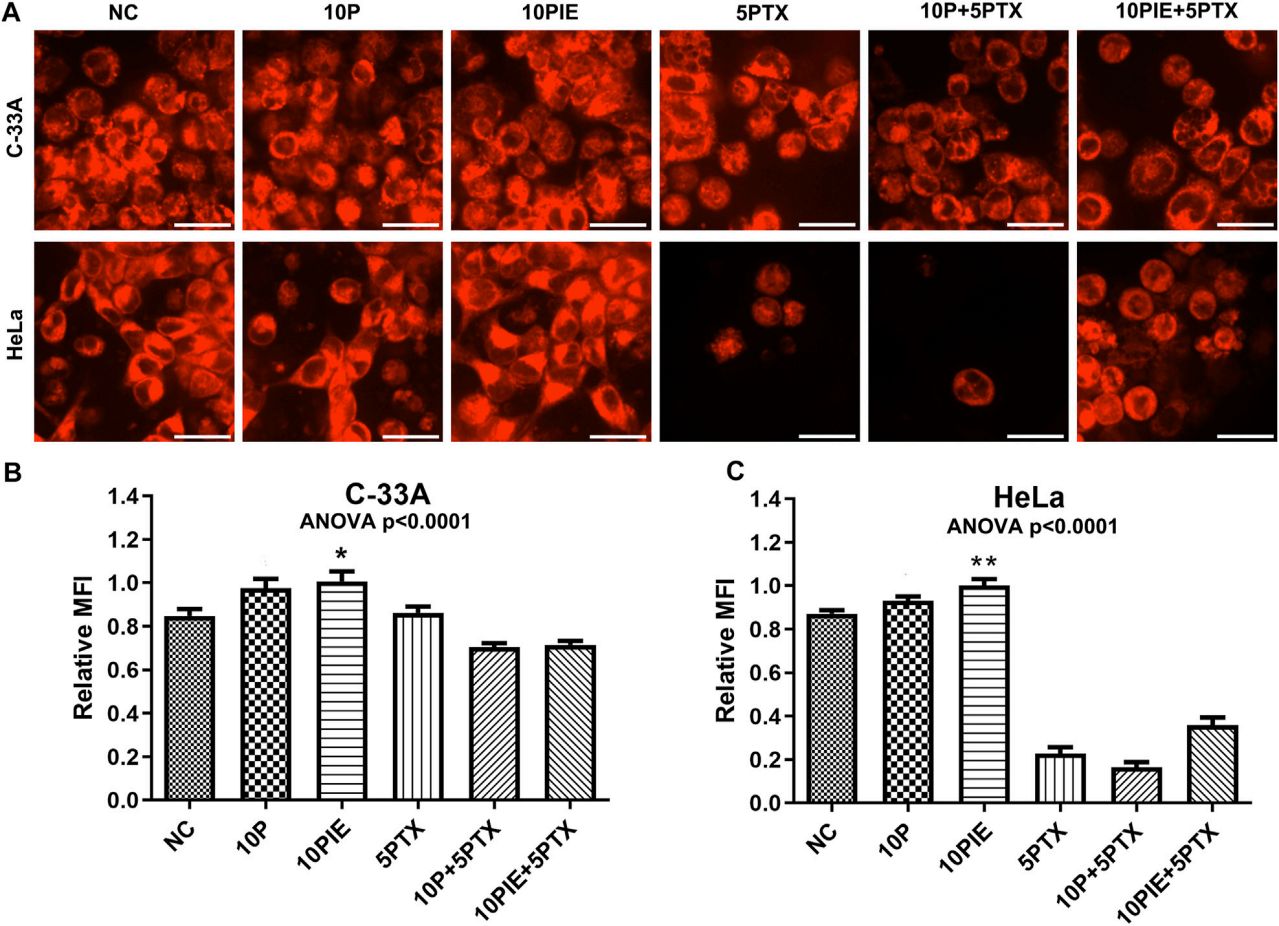
PIE increased intracellular relative MFI of ferrous ions in C-33A and HeLa cells. **(A)** Intracellular ferrous ions were detected via laser scanning confocal microscopy. All the bars indicated 20 µm. **(B,C)** Statistical analysis of intracellular MFI in C-33A and HeLa cells under different drug treatments. *p < 0.05, **p < 0.01.

To assess Fe^2+^ concentrations in C-33A and HeLa cells, we detected the intracellular MFI of ferrous ions via laser scanning confocal microscopy. Fluorescent images of intracellular Fe^2+^ in C33-A and HeLa cells treated with indicated drugs were shown in Figure 5A.

Relative MFI of ferrous ions in C-33A cells (Figure 5B) was significantly increased after treatment with 10 μg/ml PIE [(1.01 ± 0.12) vs. (0.85 ± 0.08), *p* < 0.05] compared to NC, however, 10 μg/ml propofol did not significantly increase the relative MFI compared to NC [(0.97 ± 0.12) vs. (0.85 ± 0.08), *p* = 0.10]. In the three groups involving treatment of 5 μg/ml paclitaxel, intracellular relative MFI of ferrous ions was decreased possibly due to the Fe^2+^ leakage, since apoptosis caused by paclitaxel treatment could disrupt cell membrane integrity.

Similarly, relative MFI of Fe^2+^ in HeLa cells (Figure 5C) was significantly elevated after treatment with 10 μg/ml PIE [(1.00 ± 0.10) vs. (0.87 ± 0.05), *p* < 0.01] compared to NC, however, 10 μg/ml propofol did not exert significant influence on relative MFI compared to NC [(0.93 ± 0.06) vs. (0.87 ± 0.05), *p* = 0.49]. In the three groups of treatment involving paclitaxel, intracellular relative MFI of ferrous ions was also significantly reduced.

These findings reveal that PIE treatment could influence intracellular Fe^2+^ accumulation.

### Propofol Augments Paclitaxel-Initiated Cell Ferroptosis by Regulating SLC7A11/GPX4 Pathway

To explore possible mechanisms for propofol/PIE individually or in combination with paclitaxel triggering ferroptosis-related changes in C-33A and HeLa cells, a key pathway involved in ferroptosis were examined firstly.

**FIGURE 6:**
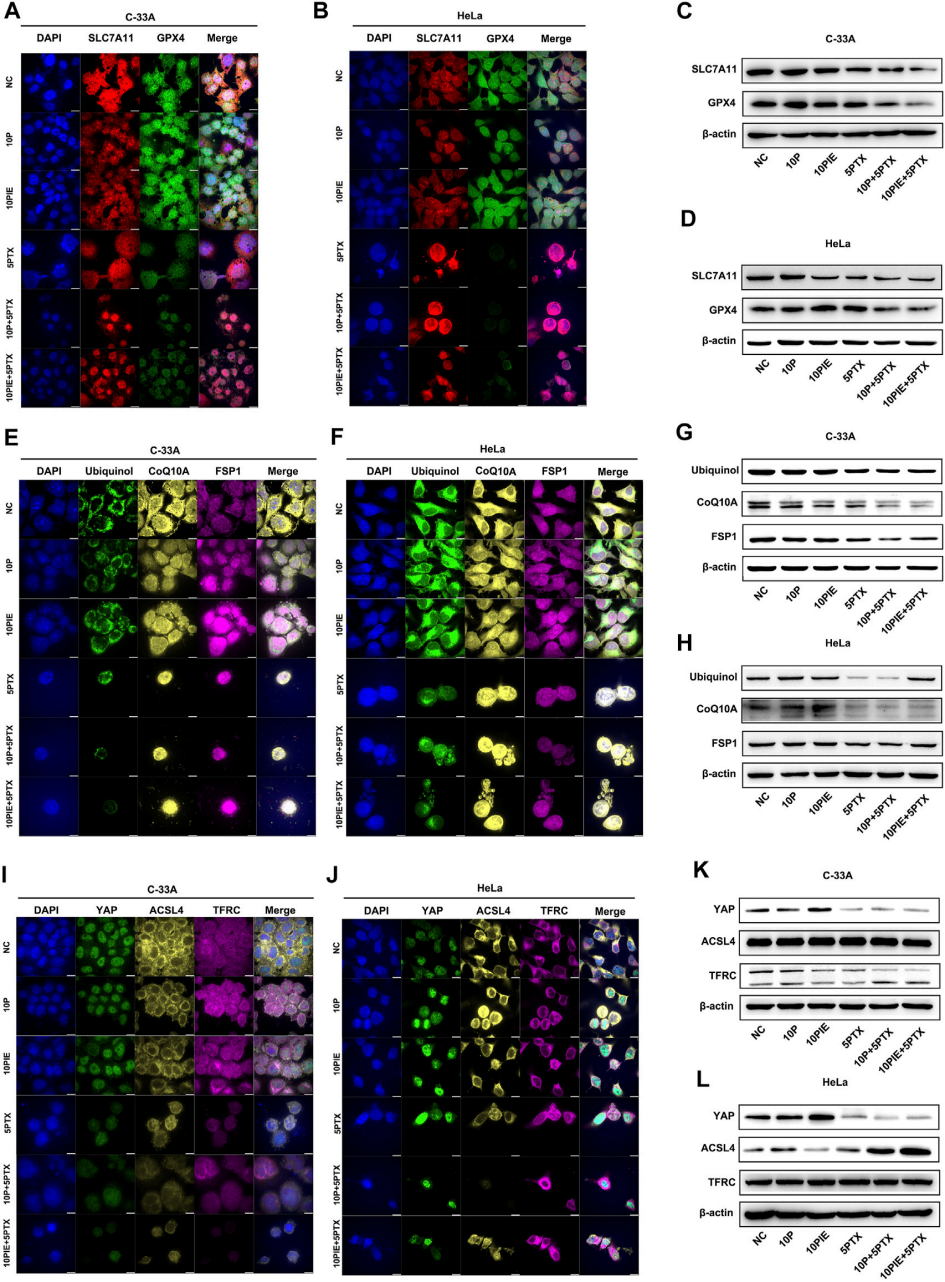
Effects of propofol or PIE with or without paclitaxel on SLC7A11/GPX4, ubiquinol/CoQ10A/FSP1, and YAP/ACSL4/TFRC signaling pathways. The expression and subcellular localization of SLC7A11/GPX4 **(A,B)**, ubiquinol/CoQ10A/FSP1 **(E,F)**, and YAP/ACSL4/TFRC **(I,J)** in C-33A and HeLa cells were detected by mIHC. The expression of SLC7A11/GPX4 **(C,D)**, ubiquinol/CoQ10A/FSP1 **(G,H)**, and YAP/ACSL4/TFRC **(K,L)** in C-33A and HeLa cells were determined by WB. All the bars indicated 10 µm.

As shown in Figures 6A,B, in C-33A and HeLa cells, SLC7A11 was mainly localized on the membrane. While GPX4, another essential ferroptosis inhibitor, exerts its effect by catalyzing the reduction of lipid peroxides, was expressed both in mitochondrion and cytoplasm as indicated.

As shown in Figure 6C, the expression level of SLC7A11 in C-33A cells was notably diminished after propofol plus paclitaxel, or PIE plus paclitaxel treatment compared to that of paclitaxel single treatment. The expression of GPX4 in C-33A cells was distinctly reduced after propofol plus paclitaxel or PIE plus paclitaxel treatment compared to that of paclitaxel treatment alone.

Comparably, in HeLa cells (Figure 6D), the expression level of SLC7A11 was largely decreased by PIE, paclitaxel, propofol plus paclitaxel, or PIE plus paclitaxel treatment. The expression of GPX4 in HeLa cells was exceedingly diminished by propofol plus paclitaxel, or PIE plus paclitaxel treatment compared to that of paclitaxel treatment alone.

Thus, we found that propofol/PIE alone or in combination with paclitaxel could regulate the SLC7A11/GPX4 pathway, which is reported to be correlated with ferroptosis.

These results indicate that propofol or PIE may enhance the anti-tumor effect of paclitaxel by inducing ferroptosis through the SLC7A11/GPX4 pathway.

### Propofol Enhances Paclitaxel-Initiated Cell Ferroptosis by Regulating Ubiquinol/CoQ10/FSP1 Pathway

Ubiquinol/CoQ10/FSP1 is another essential pathway regulating cell ferroptosis independent of SLC7A11/GPX4 (Doll et al., 2019; Tsui et al., 2019).

In C-33A and HeLa cells, CoQ10A, FSP1, and ubiquinol were mainly expressed in the mitochondrion and cytoplasm (Figures 6E,F).

In C-33A cells, the expression level of ubiquinol was decreased after the treatment of paclitaxel, propofol plus paclitaxel, or PIE plus paclitaxel for 24 h compared to NC. The expression of CoQ10A in C-33A cells was apparently reduced in propofol, PIE, or paclitaxel groups compared with NC. Propofol or PIE plus paclitaxel treatment also diminished the CoQ10A level compared to that of paclitaxel alone. FSP1 was dramatically downregulated in groups of propofol/PIE plus paclitaxel treatment compared to the group of paclitaxel single treatment (Figure 6G).

While in HeLa cells (Figure 6H), the expression level of ubiquinol and FSP1 was distinctly diminished by paclitaxel or propofol plus paclitaxel treatment compared to NC. The expression level of CoQ10A was evidently downregulated in groups involving treatment of paclitaxel in HeLa cells.

In summary, we found that propofol or PIE may induce or enhance paclitaxel-triggered ferroptosis in C-33A and HeLa cells via inhibiting the ubiquinol/CoQ10/FSP1 pathway.

### Propofol Augments Paclitaxel-Initiated Cell Ferroptosis by Regulating YAP/ACSL4/TFRC Pathway

The proto-oncogenic transcriptional co-activator YAP is activated by antagonizing E-cadherin-regulated intracellular Merlin–Hippo signaling, promoting ferroptosis by upregulating the expression of ferroptosis modulators ACSL4, TFRC, etc. (Wu et al., 2019).

In C-33A and HeLa cells, YAP was mainly localized in the nucleus, ACSL4 was expressed in the cytoplasm, while TFRC was localized in both cytoplasm and cell membrane as shown in Figures 6I,J.

In C-33A cells, the expression level of YAP was incredibly increased after the treatment of PIE for 24 h compared to NC. The three groups involving treatment of paclitaxel presented low expression level of YAP. The expression level of ACSL4 or TFRC showed no evident difference among the six groups in C-33A cells (Figure 6K).

The expression level of YAP showed comparable increase after PIE treatment, and also drastically decline in groups containing paclitaxel in HeLa cells. ACSL4 was notably upregulated by propofol compared to NC, and by propofol/PIE plus paclitaxel compared to paclitaxel alone. The expression level of TFRC showed no difference among six treatment groups in HeLa cells (Figure 6L).

**FIGURE 7:**
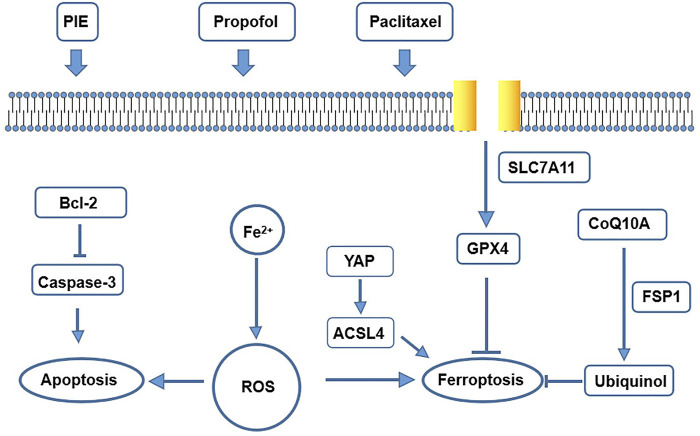
A schematic map of potential mechanisms for drug treatment leading to apoptosis and ferroptosis in our study.

In summary, we found that propofol or PIE may also have an effect on the intracellular YAP/ACSL4/TFRC signaling to promote or enhance paclitaxel-induced ferroptosis in C-33A and HeLa cells (Figure 7).

## Discussion

Ranking as the fourth leading cause of cancer death in women worldwide, cervical cancer has approximately 530,000 new cases and 275,000 deaths every year (Olusola et al., 2019). Surgery is the optimal treatment regimen for early cervical cancer. Systemic chemotherapy and radiotherapy in combination are standardized care for the most progressive cervical cancer (Olusola et al., 2019). However, recurrence rates in cervical cancer are unsatisfying. The rate of recurrence after radical hysterectomy for cervical cancer was 1.4–2.9% (Uppal et al., 2020). For metastatic, recurrent, and persistent cervical cancer, the prognosis is extremely poor, as the progression-free survival is 12.0 and 13.1 months for paclitaxel–cisplatin–ifosfamide-treated patients and paclitaxel–cisplatin–bevacizumab-treated patients, respectively (Choi et al., 2020). Therefore, targeting on new mechanisms that enhance the anticancer effect of traditional chemotherapy is urgently required. Propofol, the most widely used intravenous sedative–hypnotic agent in the operation room, has been reported to exert anticancer effects either as single treatment or as an adjuvant *in vitro*. In cervical cancer cells, propofol is able to induce cisplatin-mediated cellular apoptosis through repression of the EGFR/JAK2/STAT3 pathway (Li et al., 2017). In colorectal cancer, gastric cancer, and renal cell carcinoma, propofol directly inhibits viability, migration, and invasion of cancer cells *in vitro* (Liu et al., 2021; Shi et al., 2021; Zhao and Liu, 2021). To our best knowledge, effects of combination therapy of propofol/PIE with paclitaxel on ferroptosis of cervical cancer cells have not yet been discussed. In the current study, we are the first to report that propofol/PIE exerts synergistic anticancer effects with paclitaxel on C-33A and HeLa cells by promoting not only apoptosis, but also ferroptosis-related changes *in vitro*.

Our study found that propofol/PIE at clinical-relevant concentrations could inhibit cervical cancer cell viability *in vitro* and combination treatment of propofol/PIE with paclitaxel resulted in further suppression of cell viability. In C-33A cells, propofol and paclitaxel, PIE and paclitaxel both showed synergistic effects on the suppression of cell viability. In HeLa cells, propofol and paclitaxel had synergistic effects on inhibition of cell viability, while PIE and paclitaxel did not. Apoptosis is an extensively studied type of regulated cell death in most type of cancer cells. However, the clinical implementation of chemotherapeutic agents targeting apoptosis in oncology can be unsatisfactory due to drug resistance acquired by cancer cells. Therefore, discovering non-apoptotic RCD might provide an alternative anticancer strategy (Chen et al., 2021). The present study proved that propofol/PIE promotes apoptosis of HeLa and C-33A cells, which was consistent with previous studies (Li et al., 2017). In addition, the inhibition effects were influenced not only by apoptosis, but also by other factors such as proliferation ability or other kind of cell death, such as ferroptosis.

Furthermore, TEM tests have shown significantly more ferroptosis-related morphological changes, shrunken mitochondria, after single treatment of PIE or paclitaxel in C-33A cells. Propofol significantly enhanced paclitaxel-induced ferroptosis-related morphological changes of HeLa cells. These findings are novel as no publication has discussed the possible effects of anesthetic propofol/PIE on ferroptosis of C-33A or HeLa cells. Ferroptosis can be inducted by iron accumulation, excessive ROS, or inhibited GPX4 (Chen et al., 2021). Our results showed that PIE increased ferrous ions, propofol/PIE combined with paclitaxel enhanced intracellular ROS, and suppressed GPX4 expression in C-33A and HeLa cells. To elucidate how propofol/PIE exerted effects on ferroptosis-related changes in C-33A and HeLa cells, we detected the changes of the SLC7A11/GPX4 pathway, ubiquinol/CoQ10/FSP1 pathway, and YAP/ACSL4/TFRC pathway. We found the ferroptosis-related pathways were influenced by drug treatments (Figure 7).

In conclusion, this *in vitro* cell study suggests that propofol or PIE (the clinical anesthetic containing propofol as a major component) could be a potential adjuvant to augment chemotherapeutic sensitivity of cervical cancer cells via the ferroptosis activities. Future studies are needed to elucidate the potential mechanisms of the relationship between propofol/PIE and cancer cell ferroptosis more thoroughly.

## Data Availability

The original contributions presented in the study are included in the article/Supplementary Material; further inquiries can be directed to the corresponding authors.
